# Hydrophobic Collapse of Trigger Factor Monomer in Solution

**DOI:** 10.1371/journal.pone.0059683

**Published:** 2013-04-02

**Authors:** Kushagra Singhal, Jocelyne Vreede, Alireza Mashaghi, Sander J. Tans, Peter G. Bolhuis

**Affiliations:** 1 van ‘t Hoff Institute of Molecular Sciences, University of Amsterdam, Amsterdam, The Netherlands; 2 Department of Systems Biophysics, FOM Institute AMOLF, Amsterdam, The Netherlands; University of Leeds, United Kingdom

## Abstract

Trigger factor (TF) is a chaperone, found in bacterial cells and chloroplasts, that interacts with nascent polypeptide chains to suppress aggregation. While its crystal structure has been resolved, the solution structure and dynamics are largely unknown. We performed multiple molecular dynamics simulations on Trigger factor in solution, and show that its tertiary domains display collective motions hinged about inter-domain linkers with minimal or no loss in secondary structure. Moreover, we find that isolated TF typically adopts a collapsed state, with the formation of domain pairs. This collapse of TF in solution is induced by hydrophobic interactions and stabilised by hydrophilic contacts. To determine the nature of the domain interactions, we analysed the hydrophobicity of the domain surfaces by using the hydrophobic probe method of Acharya *et al.* [1], [2], as the standard hydrophobicity scales predictions are limited due to the complex environment. We find that the formation of domain pairs changes the hydrophobic map of TF, making the N-terminal and arm2 domain pair more hydrophilic and the head and arm1 domain pair more hydrophobic. These insights into the dynamics and interactions of the TF domains are important to eventually understand chaperone-substrate interactions and chaperone function.

## Introduction

Most proteins, synthesised as linear polypeptide chains in ribosomes, have to fold into specific and unique 3-D structures in order to function. In complex cell environments, the spontaneous unassisted folding process is highly prone to misfolding, leading to the formation of dysfunctional proteins and aggregates [3]. Molecular chaperones suppress these anomalies by interacting with the newly synthesised proteins by stabilising proteins in the cytosol or by maintaining the unfolded polypeptide chains for translocation through organelle membranes [4]–[8]. These functions are performed through non-covalent interactions between proteins (or polypeptide chains) and chaperones, either at the ribosome-exit tunnel or in the cytosol [9].

Trigger factor (TF) is a chaperone characterised in bacteria (e.g., *E. coli*) and chloroplasts [10], [11]. Unlike many molecular chaperones, TF does not need ATP to function. It is located in the cytosol as well as near the ribosome exit tunnel. At the ribosome exit tunnel, the chaperone is coordinated via a glutamic acid residue on ribosomal protein L23 [12], and is thought to interact with most polypeptides early during their synthesis to assist in their folding [13]–[18]. In the cytosol, TF is found in a dimer-monomer equilibrium [19]–[24] and supposedly interacts with full-length proteins in order to prevent aggregation [24], [25].

**Figure 1:**
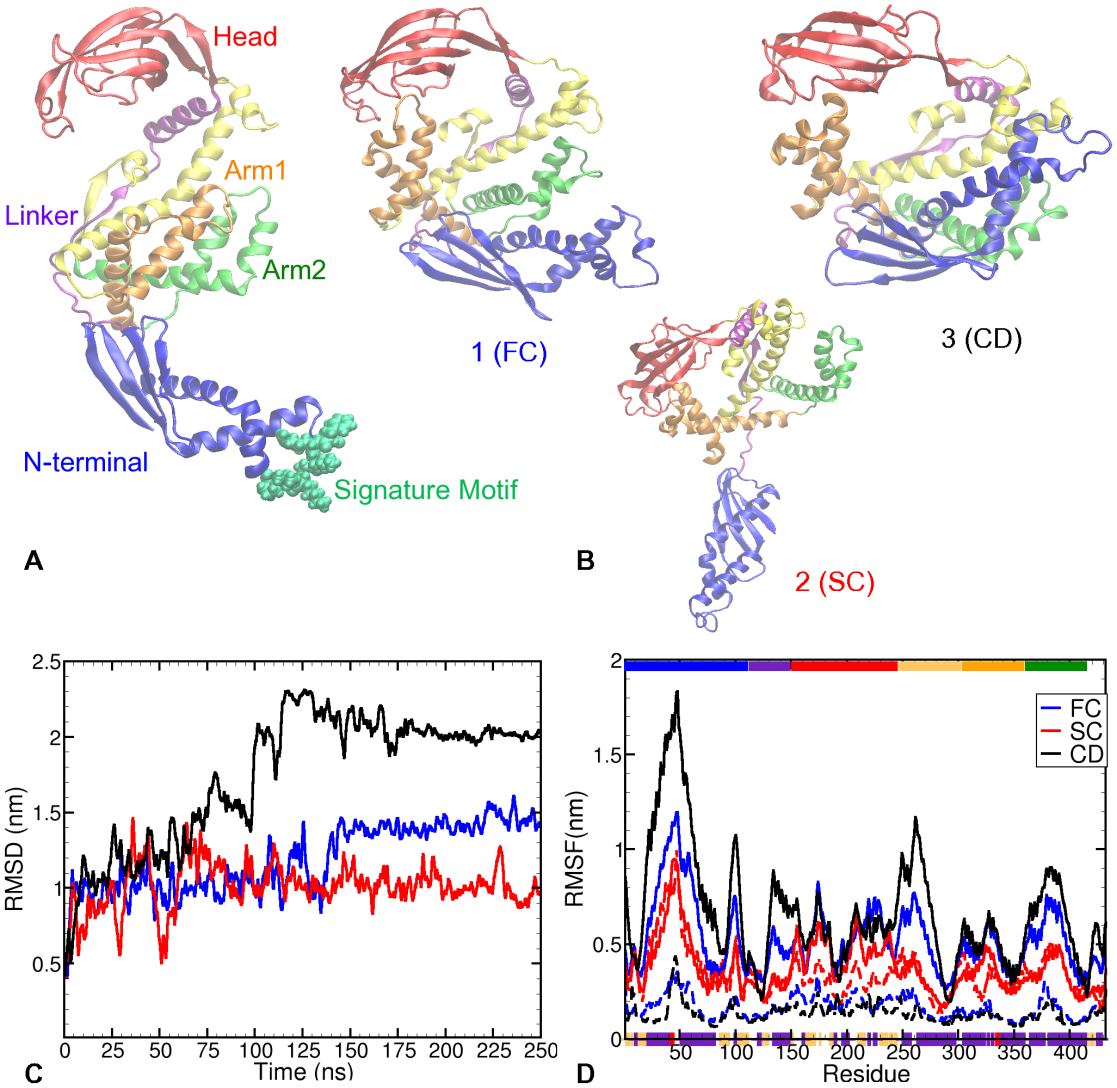
Flexibility of Trigger factor. (**A**) Crystal structure of Trigger factor monomer (1W26): N-terminal is shown in blue; PPIase domain (Head) shown in red; C-terminal is shown in yellow, with the two arm-like extensions – Arm1 and Arm2 - shown in orange and green respectively. The long helix connecting head and arm1 is shown in yellow and named Head-Arm1 linker. The signature motif that binds TF to the ribosome is illustrated in green beads [11]; (**B**) The 3 resulting structures corresponding to the 3 representative trajectories (same colour coding as (A)), obtained after 250 ns long simulations; (**C**) Structural deviations of Trigger factor over time are quantified through average root-mean square deviations (RMSD) of C-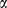 atoms from their respective positions in the crystal structure. The plot shows 3 representative RMSD time evolutions: 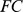 represents 6 trajectories with significant deviations from the crystal structure, 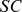 is representative of 5 trajectories with the least deviations from the crystal structure, while 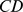 represents 1 trajectory with the highest deformation; (**D**) Time-averaged root mean square fluctuations (RMSF) plotted for each amino acid residue. The solid lines represent the fluctuations over the complete trajectory, while the dashed lines represent the structural fluctuations in the last 20 ns of the respective trajectories. Graphs 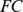, 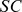 and 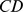 are averaged over the trajectories that they represent. The secondary structure elements corresponding to the residues are plotted on the bottom x-axis: indigo blocks represent 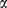-helices, ocher blocks 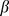-strands, and red blocks 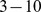 helices. The tertiary structure is represented at the top x-axis as blocks that colour-coded in the same way as in (A).

Figure 1(A) shows the crystal structure (PDB code 1W26) of Trigger factor [20]. With 432 amino acid residues, the crystal structure of this 48 kDa protein adopts an elongated dragon-shaped conformation, containing three distinct tertiary domains, viz. N-terminal, PPIase (Peptidylprolyl isomerase), and C-terminal domains [11], [20], [26], [27]. The N-terminal domain of TF (residues 1–149) forms the “tail” of the dragon, and contains the signature-motif “GFRxGxxP” at residues 43–50 that mediates ribosome docking [12], [28]. The core (residues 1–111) of N-terminal is connected to the PPIase domain (residues 150–245), which forms the head of the dragon, through a linker (residues 112–149). The third domain, called C-terminal domain (residues 246–432), is postulated to be responsible for the chaperone function of TF [29]. The C-terminal forms the rest of the body of the dragon and consists of two arm-like extension loops, that are termed as arm1 (residues 303–360) and arm2 (residues 361–415), respectively. The long 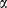-helix in the C-terminal domain between residues 246 and 302, which links head with arm1, is labeled “Head-Arm1 Linker” or HA1-linker. Comparison studies of different free and substrate-bound structures have indicated that TF can adopt different structures [20], [24], [28], [30], [31], hinting that it is flexible, with hinge-bending motions in C-terminal [11]. The flexibility is defined in terms of the rotational freedom of its PPIase domain about the HA1-Linker (estimated at ∼25° [24], [31]) and flexion of N-terminal to C-terminal domain (estimated at ∼10° in case of *T. maritima* [24]), and that of C-terminal core to its arms (estimated at ∼25° with slight additional local flexibility in the two arms [20], [24], [28], [30], [31]).

Cross-linking experiments, and refolding and reactivation studies on a dehydrogenase in absence and presence of TF have confirmed that TF displays *in vitro* chaperone activity in preventing aggregation and promoting refolding of denatured model substrates [32]–[36]. The optimum molar ratio of TF to protein substrates has been determined to lie between 1∶1 to 4∶1 for its functioning [32], [35]. Maier *et al.* demonstrated that at higher concentrations (40∶1), TF efficiently prevents aggregation, but tends to decrease the refolding yield of protein substrates [34]. However, there is no conclusive explanation for its role in the folding process of its substrates. Hoffman *et al.* [11] have proposed that as the only ribosome–associated chaperone in *E. coli*, TF can assist folding in two mutually non-exclusive ways: by keeping nascent chains rather unfolded and, thus, preventing misfolding during synthesis [37]; or by directly accelerating productive co-translational folding processes.

Due to crystal contacts in a lattice, the structure of TF in crystal is expected to be different from that in solution [30]. The characterisation of the structure of TF in a solution is, thus, a pre-requisite to understanding the mechanics of TF in the cytosol. As TF is too large for structural determination by NMR spectroscopy, molecular simulations can play an important role in gaining an understanding of the conformations of TF monomer in solution. O’ Brien *et al.* have performed coarse-grained simulations of the interaction between nascent polypeptide chains with ribosome-bound TF [38]; however, the structure of monomeric TF in solution is still unexplored in simulations. Here, we aim to gain such insight by performing large scale all-atom molecular dynamics (MD) simulations on the conformational changes of TF in explicit solvent. Due to the size and elongated shape of TF, MD simulations in explicit solution are computationally challenging. While we cannot expect the MD to completely sample the conformational space of TF, our simulations clearly show that the chaperone deviates from its crystal structure, is highly flexible and displays large fluctuations. Paradoxically, this enhanced flexibility allows TF to collapse into a more compact structure in solution. We show that the collapse is driven by hydrophobic contacts, and further stabilised by hydrophilic contacts. The hydrophobic character of these contacts are not directly given by the standard hydrophobicity scale determined by the individual residues since hydrophobicity is a collective phenomenon. To investigate the effective nature of the contacts, we employed the method of Acharya *et al.* [1], [2] that estimates hydrophobicity of surface residues by the affinity of probe particles. The resulting effective hydrophobicity map of the TF surface is found to be very different from the individual standard hydrophobic map and is dependent on the conformation. The hydrophobicity maps characterised in different conformations of TF result in a better understanding of the interactions occurring in the collapse process, and are ultimately required to understand the chaperone function of TF.

## Results and Discussion

We performed twelve MD runs, each 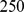 ns long (with different initial velocities) on an isolated monomeric TF chaperone in 50 mM NaCl-solution. Interactions in eight of these trajectories were defined by the AMBER03 forcefield [39], and by the OPLS-AA forcefield [40] in the other four. In addition, we performed some simulations under different conditions with GROMOS43a1 [41], [42] (see File S1). On an IBM Power 6 machine each trajectory run with OPLS-AA parameters (system size: 205000 atoms, time step: 2.0 fs) took a total of 432 CPU hours on 128 cores (total of 55232 core hours per trajectory); each trajectory run with AMBER03 parameters (system size: 165000 atoms, time step: 2.0 fs) took 525 hours on 64 cores (total of 33284 core hours); each trajectory run with AMBER03 parameters (system size: 165000 atoms, time step: 1.5 fs) took 761 hours on 64 cores (45488 core hours). These simulations produced more than 1 TB of data for further analysis. While a 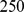 ns long simulation is computationally demanding for this system size, it is not sufficient to ergodically sample the configurational space of the TF monomer. Despite this shortcoming, the resulting trajectories give a good indication of the relaxation of TF towards a (local) equilibrium, thus giving insight into the behaviour of TF in solution. When using different force fields we naturally expect differences in the distributions of the population, in the loss in secondary structure (specifically, 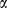-helical stability) and the strength of salt-bridges (both higher in case of OPLS-AA and GROMOS43a1 compared to AMBER03). Nevertheless, in general different force fields result in remarkably robust structure and dynamics [43].

### Collapse of Trigger Factor

Visual inspection of all trajectories show a substantial structural change of the chaperone molecule in solution, indicating that the crystal structure is not stable in solution. The simulations show that starting from an extended crystal structure conformation, TF collapses and becomes more compact in solution. Based on the observed structural evolution we can group the 12 trajectories into three different sets, labeled 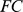 (fully collapsed), 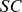 (semi-collapsed) and 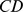 (collapsed and deformed). Figure 1(B) shows three conformations from three trajectories representing these sets, acquired after 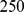 ns of MD simulation in solution. The time evolution of the deviation from the crystal structure is quantified by plotting the root mean square deviations (RMSD) of C-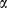 atoms with respect to the crystal structure in Figure 1(C). The RMSD plots of all trajectories are shown in Figure S1 in File S1.

Trajectory 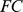 represents a full collapse, as observed in six out of twelve trajectories. These trajectories lead to conformations such as structure 1 shown in Figure 1(B) – a collapsed conformation with head and arm1 stacked together as well as N-terminal and arm2 stacked together. In the rest of the paper we will refer to the domain pair of head and arm1 as 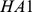 and that of N-terminal and arm2 as 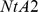. One out of twelve trajectories exhibits a larger RMSD, representing a collapse that results in the structure 3 shown in Figure 1(B). This trajectory is labeled 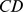 for the collapse and further deformation in the TF’s structure via interactions between N-terminal and long helix of C-terminal. In addition to the formation of domain pairs (similar to structure 1), structure 3 also exhibits an interaction between N-terminal and HA1 Linker. Conversely, trajectory 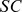 (semi-collapse), representing the other five trajectories, shows an RMSD of ∼1.0 nm. These trajectories end in a semi-collapsed conformation, exhibiting only the HA1 collapse, while N-terminal is stretched away from arm2 (see Figure1(B)). These five trajectories comprise of all four trajectories run with the OPLS-AA forcefield parameters, and one with the AMBER03 parameters. Note that while the representative trajectory was run with AMBER03 parameters, those performed with OPLS-AA parameters show comparable behaviour. Thus, while the HA1 collapse is predicted by both forcefields, the OPLS-AA forcefield does not sample the NtA2 collapse within the time frame of our simulations.

Figure 1(D) presents time-averaged root mean square fluctuations (RMSF) per amino acid residue for the three trajectory sets. Note that this time-averaging does not imply ergodicity. We perform this time-average only to conveniently present the dynamical behaviour of each amino-acid residue through the trajectory. The graphs of 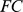 and 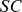 are averaged over the trajectories that they represent. We qualitatively compared these RMSF measurements to the NOE measurements conducted by Yao *et al.* [30] on a construct of residues 113–432
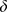
150–245. In their work, Yao *et al.* identified regions at residues 130–140, 141–150, 365–385, 415–423, as well as certain residues around residue 320 as flexible. In our RMSF plots, the largest fluctuations are observed in the core of the N-terminal (residues 20–70) as indicated by the peak at the signature motif loop (residues 43–50). As expected, the fluctuations observed in the N-terminal and in arm2 (residues 365–395) are higher for trajectories 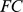 and 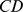 than those in 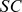. Additionally, 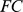 and 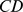 also demonstrate high fluctuations in the inter-domain linkers, namely, the linker between N-terminal and PPIase domain (residues 130–149, the purple coloured helical region in Figure 1(A)), and residues 246–276 (shown in yellow in 1(a)) forming a combination of a loop starting at the end of PPIase domain and part of the long helix that makes up rest of HA1 linker. These linker regions show much larger fluctuations in the case of 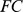 and 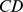 than in case of 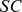. This difference becomes even more striking when comparing the fluctuations of these linkers relative to the average fluctuation in the protein. The 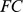 and 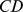 trajectories show clearly a relatively larger fluctuation in the linker regions. These large structural fluctuations in linker and loop regions are in agreement with the literature [11], [20], [28], [31], and form part of the flexibility that TF exhibits in its extended structure.

After the collapse and formation of domain pairs, the fluctuations in TF are expected to be significantly attenuated. The structural fluctuations in the last 20 ns of the three representative trajectories are shown as dashed lines in Figure 1(D). For trajectories 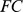 and 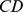, structural fluctuations are uniformly attenuated by approximately a factor of 5. In contrast, fluctuations in 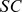 are almost independent of the time window, except for the collapsed pair of domains (HA1), which shows a slight attenuation in fluctuations over the last 20 ns. This suggests that TF does not lose its flexibility in the semi-collapsed trajectory. In the 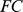 trajectory, the subduing of structural fluctuations after the collapse of both HA1 and NtA2 domain-pairs indicates that the fully collapsed state is rather stable, at least on the timescale of the simulations.

During the collapse processes, the number of helical hydrogen bonds in TF does not change significantly over time, fluctuating between 140 and 150. The secondary structure elements of TF remain unperturbed in solution, in agreement with the observations of Yao *et al.* [30]. Further, the NMR study by Yao *et al.* showed that residues K127–I129 form a parallel 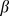-sheet with residues T418–T422. We observe that, in our simulations, this parallel 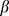-sheet structure is conserved despite the collapse and formation of a structure more compact than the X-ray structure of TF (see Figure S3 in File S1). The stability of the secondary structure combined with high structural fluctuations in the linker elements of TF suggests that the conformational changes consist of collective rigid-body motions of tertiary domains, viz. head, N-terminal, arm1 and arm2, with the linker elements acting as hinges (see also Figure S2 in File S1).

**Figure 2:**
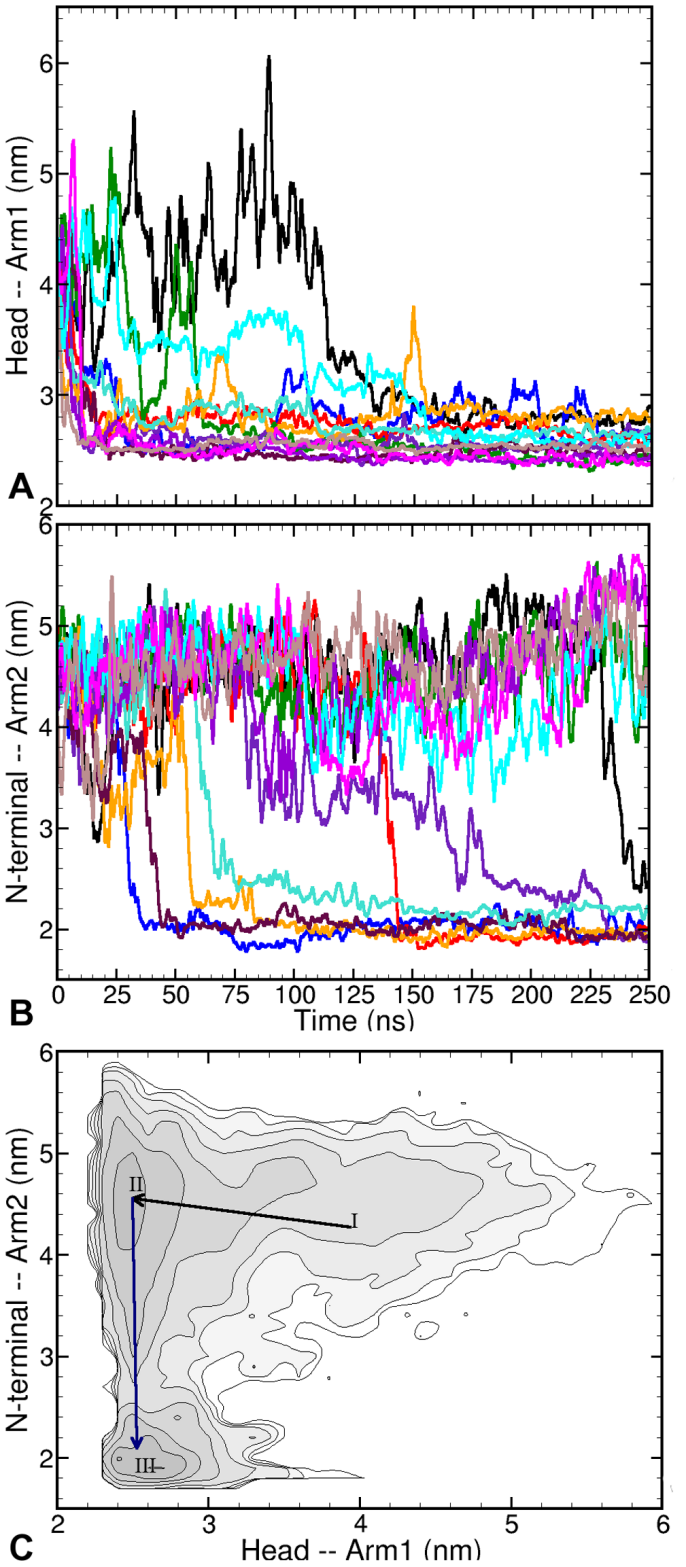
Collapse of the Trigger Factor. Time traces of pair-wise centre of mass distances between: (**A**) Head and Arm1, and (**B**) N-terminal and Arm2, show that in nearly all the trajectories the respective separation between pairs of domains is consistently reduced; (**C**) Logarithmic probability histogram plot for pair-wise COM distances – HA1 vs NtA2– shows three regions corresponding to: initial extended structure (I), semi-collapsed collapsed (II), and the fully collapsed. Contours are 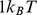 apart. Note that this plot does not represent an equilibrium free energy landscape, as the simulations are not converged.

Figures 2(A) and 2(B) present this domain-pair formation in terms of change in inter-domain centre of mass (COM) distances – HA1 and NtA2. The NtA2 distance relaxes to approximately 2 nm for 7 out of 12 trajectories (except in the 5 trajectories represented by trajectory 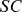). Similarly, the HA1 distance decreases in all trajectories to stabilise at ∼2.6 nm. Thus, the collapse of TF in solution consistently reduces the pair-wise distances between respective domains to comparable values in all trajectories. Comparison between Figures 2(A) and 2(B) reveals the sequence of conformational changes of TF, which starts with the HA1 collapse, followed by stacking of N-terminal and arm2 together. The first process starts on average after 25.1 ns and takes around 18 ns to complete. The NtA2 collapse takes roughly 25 ns but starts almost 49 ns after the completion of the HA1 collapse. Summarising this sequence of events in terms of the collapse of the two pairs - HA1 and NtA2, Figure 2(C) shows a 2-dimensional (logarithmic) population distribution (or probability histogram) plot of the COM distances between pairs. (We note again that this time averaged plot does not imply ergodic sampling, and cannot give a complete picture of the free-energy landscape of TF. Instead it should be read as a convenient summary of the simulation data.) The first region (I) corresponds to the extended initial structure of TF with large inter-domain distances. A minimum (region II) is observed at a short distance between head and arm1 (2.6 nm) and an almost unchanged distance between N-terminal and arm2 (∼4.5 nm), representing the semi-collapsed conformation. The global minimum (region III) in this plot corresponds to the collapsed conformation, and is observed when the distance between N-terminal and arm2 is approximately 2.0 nm and that between head and arm1 is 2.6 nm. The histogram suggests that both the semi-collapsed and the collapsed conformations are metastable on the timescale of the simulations.

While in principle the observed collapse could be an artifact of the used water model (TIP3P), this is not likely, as the collapse behaviour is also seen for the SPC water model using the GROMOS43a1 force field (see Figure S4 in File S1). Moreover, an additional 150 ns run employing OPLS-AA with the TIP4P model showed a stable collapsed structure (data not shown).

### Nature of Interactions in Trigger Factor

**Figure 3:**
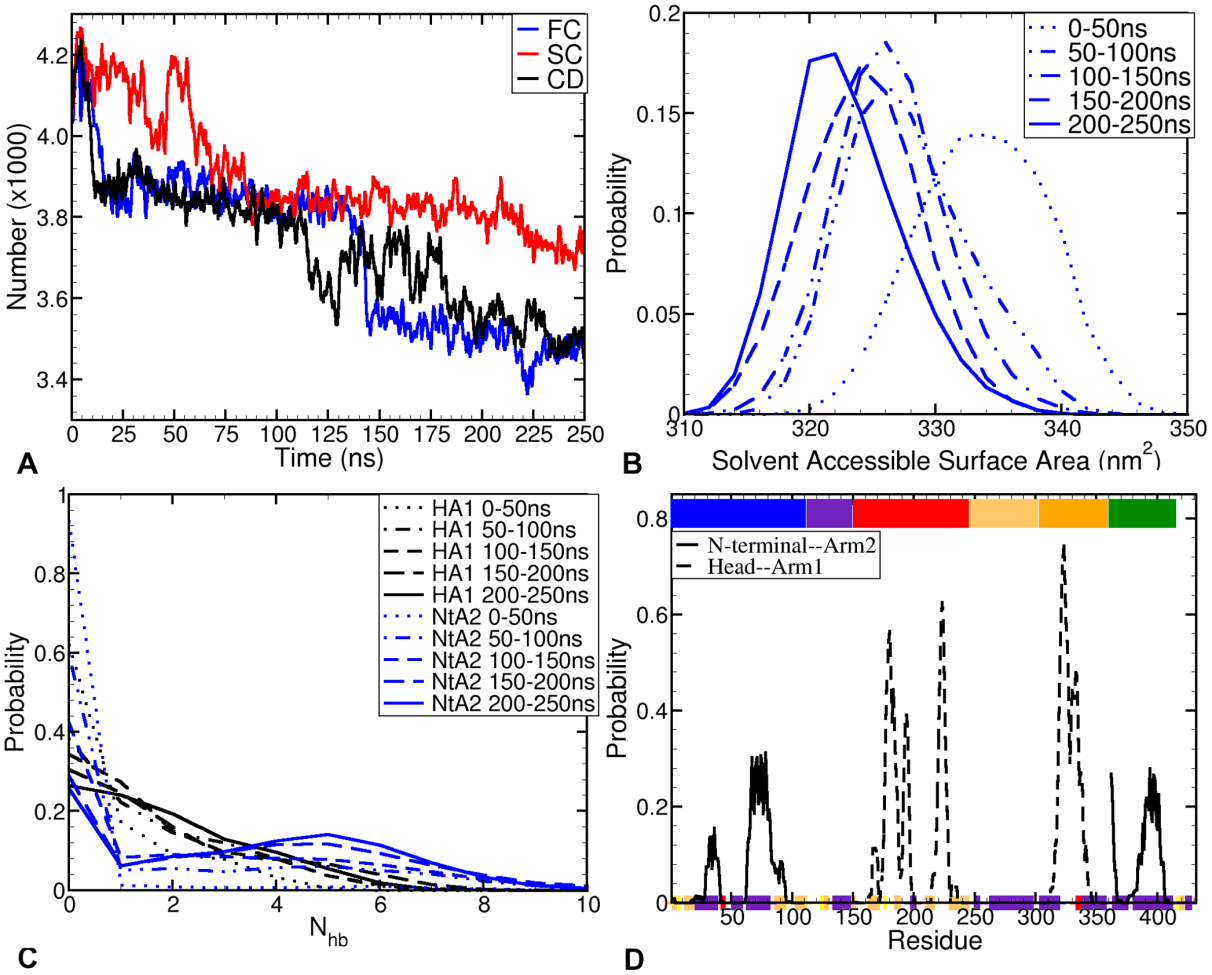
Solvation and contacts in Trigger factor. (**A**) Number of water molecules in a shell of 0.6 nm radius around Trigger factor, for the 3 representative trajectories - 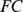 (blue), 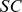 (red) and 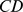 (black). There is a distinct drop in the water shell density with time, consistent with the respective HA1 and NtA2 collapses in the trajectories. (**B**) Histogram plot of the solvent-accessible surface area of TF in five consecutive 50 ns time-windows of MD simulations, suggesting that the collapse is accompanied by a burial of hydrophobic patches into the interior, more so in case of the fast HA1 collapse than in the NtA2 collapse. To investigate the second drop in SAS, we used the seven trajectories represented by trajectories 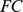 and 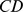. (**C**) Histogram plot of the number of pair-wise hydrogen bonds in five different 50 ns long time-windows of the MD simulation trajectories. The distribution of hydrogen bonds between HA1 is shown in blue lines, while that between NtA2 is shown in black lines. In both domain pairs, the probability of a greater number of hydrogen bonds increases with time (as the collapse is stabilised). At the same time, the latter pair (N-terminal and arm2) displays a distinctly higher propensity to form more hydrogen bonds than the former; (**D**) Probability graph of formation of contacts between corresponding domains - NtA2, and HA1. The solid black line depicts the probability of residues forming an NtA2 contact, while the dashed line shows the same for HA1 contacts. The secondary and tertiary structure elements are represented in the same way as Figure 1(D).

Figure 3(A) shows the number of water molecules in a 0.6 nm shell around TF over time in the three representative trajectories (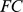, 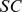, and 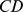). All three trajectories show a significant loss in the water density over time, with a more pronounced drop of ∼20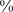 in trajectories 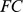 and 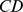. These changes in the water structure around TF are attributed to the HA1 and NtA2 collapses. In all three trajectories, the sudden decrease in the number of water molecules from 4200 to 3800 corresponds to the fast HA1 collapse; whereas a further drop from 3800 to 3200, seen only in 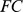 and 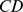, corresponds to the NtA2 collapse. This drop in the number of solvent molecules is related to a decrease in the solvent-accessible surface (SAS) of TF due to the burial of residues. Figure 3(B) shows probability histograms of TF’s SAS area over 5 consecutive 50 ns windows along the MD trajectories. The histograms were calculated over the 7 trajectories that demonstrate a full collapse, i.e., the 7 trajectories represented by 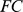 and 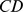. The distributions shifts to lower values over time, though the magnitude of the shift varies over windows. The most significant shift, observed between the first 50 ns and the next, is largely associated with the fast HA1 collapse. Similarly, while the HA1 collapse plays a minor role in subsequent peak shifts, they are chiefly associated with the gradual NtA2 collapse.

#### Inter-Domain contacts

Figure 3(C) presents the (logarithmic) probability distribution of the number of inter-domain hydrogen bonds (N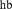) in TF. The distribution is plotted for five consecutive 50 ns windows along the MD trajectories. In both pairs - viz. HA1 and NtA2, the average number of hydrogen bonds increases with time, as the collapsed state is stabilised. Note that the distribution is broad owing to the large interval of averaging, and heterogeneity of interactions. This implies that the formation of domain pairs during both collapse processes is accompanied by the establishment of hydrophilic contacts. The number of hydrogen bonds is higher in the NtA2 collapse. The interaction between head and arm1 involves only a few hydrogen bonds; the interaction between N-terminal and arm2 comprises of a larger number of hydrophilic contacts. Figure 3(D) shows the contact probability of residues on the tertiary domains, i.e., the probability that a residue pair on corresponding domains – NtA2 and HA1– has a minimum distance of less than 0.4 nm. The solid line is for the residues in NtA2 contact, while the dashed line is for residues in HA1 contact. Tables 1 and 2 list the most likely occurring contact residue pairs involved in these inter-domain contacts. All contacts expected to be hydrophobic are italicised. The list of interacting pairs of residues in HA1 collapse in Table 1 is dominated by strongly hydrophobic residues. A significant number of these contacts can be identified as distinctly hydrophobic – (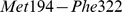), (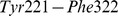), and (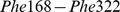). We also observe strongly hydrophobic residues (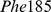, 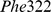, 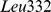 and 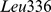) forming multiple contacts with Glycine residues and hydrophilic residues with long aliphatic sidechains (Glutamine, Lysine). These contacts are expected to be of hydrophobic character, and are italicised in the table. At first sight, it seems strange that the 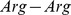 pair is the most abundant contact. However, their long partly hydrophobic side-chains are able to form a hydrophobic contact [44]. Additionally, owing to the neighbouring residues, the contact 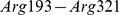 is rendered hydrophobic. Some residues – especially 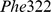, 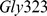, 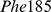, and 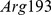 – occur multiple times in different residue-pairs, suggesting the importance of these residues in the formation of HA1 interface.

**Table 1:** Contacts between Head and Arm1.

Head	Arm1
*Arg193*	*Arg321*
*Phe185*	*Gly323*
*His222*	*Leu336*
*Arg193*	*Phe322*
*His222*	*Leu332*
*Met194*	*Phe322*
*Tyr221*	*Phe322*
*Met194*	*Gly323*
*His222*	*Phe322*
*Phe168*	*Phe322*
Arg193	Gly323
*Phe185*	*Gly324*
Gly179	Asn325
Arg193	Gln320
*Phe185*	*Gln320*

List of amino-acid residue pairs involved in inter-domain interactions between Head and Arm1, arranged in descending order of occurrence. Contacts expected to be hydrophobic are italicised.

**Table 2:** Contacts between N-terminal and Arm2.

N-terminal	Arm2
*Arg73*	*Arg399*
*Ile76*	*Arg399*
Asp69	Lys392
Ser72	Arg399
*Ile80*	*Leu360*
*Ile80*	*Lys361*
Asp69	Arg399
Gly68	Lys392
*Ile80*	*Ala362*
Asp65	Lys392
Ser72	Asp396
Asp77	Lys361
Asp65	Lys390
Arg73	Glu364
*Asp69*	*Met395*
*Ile80*	*Leu403*
Glu31	Lys390

List of amino-acid residue pairs involved in inter-domain interactions between N-terminal and Arm2, arranged in descending order of occurrence. Contacts expected to be hydrophobic are italicised.

In contrast to the HA1 interaction, the list of amino-acid residues involved in the NtA2 collapse (shown in Table 2) is dominated by charged polar residues like Glutamate, Lysine, Arginine and Aspartate, forming salt-bridges (and hydrogen bonds). There are numerous hydrophobic contacts, primarily formed by 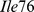 and 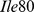 (of N-terminal) with hydrophobic residues as well as aliphatic parts of charged residues in arm2. Residues 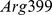 and 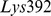 on arm2 form multiple contacts with 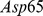 and 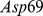 on N-terminal. The lists of contact-pairs supports the hypothesis that while hydrophobic contacts play an important role in both collapse processes – especially in the fast HA1 collapse – the stabilisation of NtA2 collapse is achieved via mainly hydrophilic contacts.

#### Characterisation of hydrophobicity

**Figure 4:**
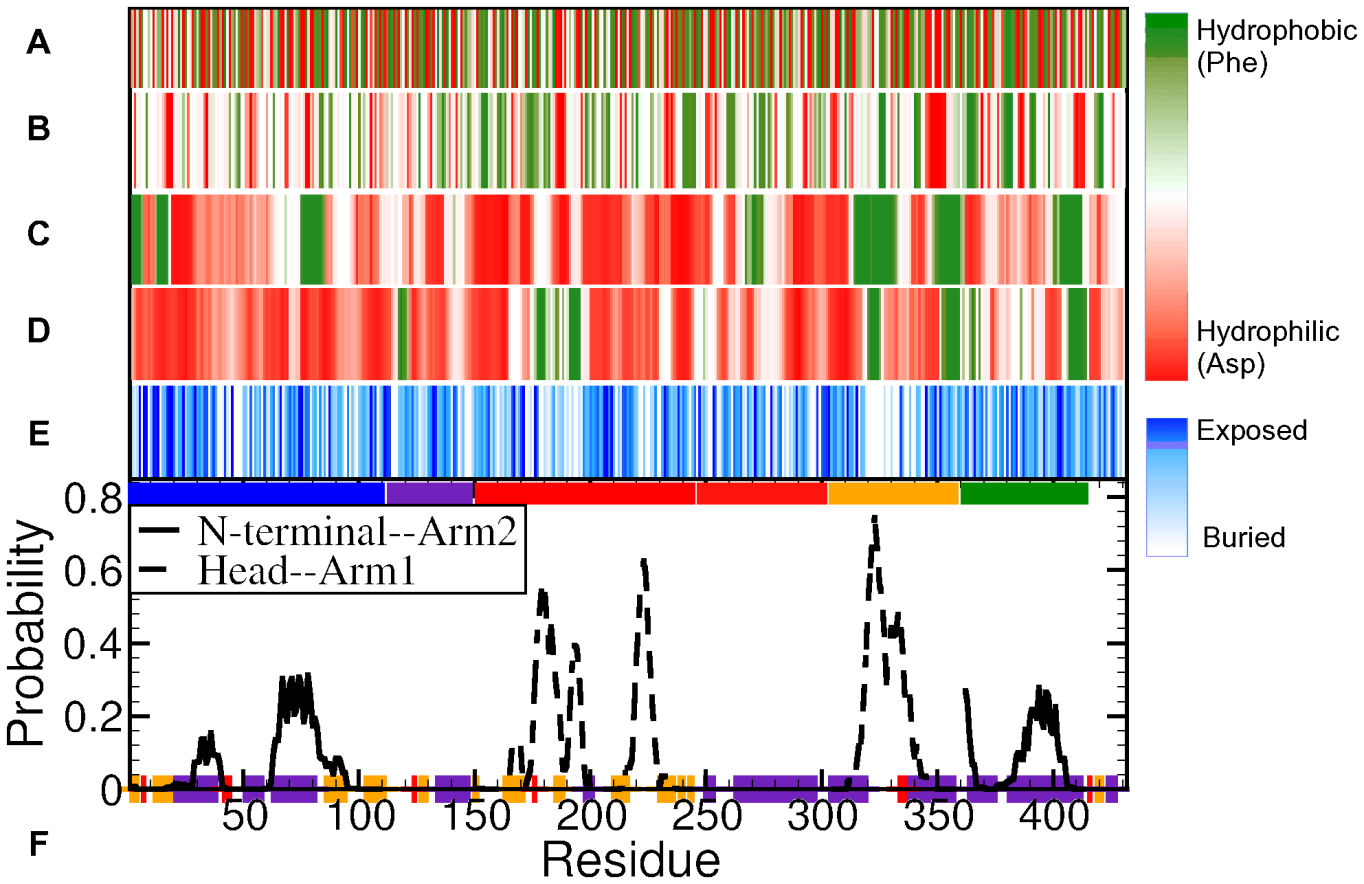
Hydrophobicity of Trigger factor. (**A**) Standard hydrophobicity of TF per residue [48], plotted as a coloured-barcode. The hydrophobicity scale varies from red – very hydrophilic (viz. 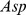) – to green – very hydrophobic (viz. 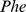). White-coloured residues are more likely to be hydrophobic than hydrophilic. (**B**) Standard hydrophobicity of TF residues, sequentially-averaged over groups of 5 residues. On the same hydrophobicity scale, hydrophobicity maps obtained from averaged (over 4 MD runs of 30 ns each) probability histograms of binding of hydrophobic probes to TF are also plotted, (**C**) in the extended conformation, (**D**) in the collapsed conformation (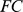) from Figure 1(B). The graphs are averaged over sequential groups of 5 residues. (**E**) Barcode representing the extent of burial (white) and exposure (blue) of all Trigger factor residues upon collapse. (**F**) These plots are overlaid on the probability graph of formation of contact pairs in NtA2 and HA1 collapses, as in figure 3(D).

The conventional way of detecting hydrophobic patches is by assigning a hydrophobic/hydrophilic measure to each amino acid, as shown in figure 4(A). The magnitude of hydrophobicity is shown on a red-white-green (RWG) scale – green being the most hydrophobic (viz. Phenylalanine) and red the most hydrophilic (viz. Aspartate). In this standard map, the hydrophobic residues are scattered randomly along the protein sequence. In figure 4(B) we show the hydrophobicity along the sequence averaged over 5 consecutive residues. However, as the hydrophobic effect is a collective phenomenon, the hydrophobic character of a group of residues cannot be accurately determined by that of the individual residues. To investigate the hydrophobic character of TF, we characterised group of residues displaying collective hydrophobic behaviour using the hydrophobic probes method [1], [2]. Developed by Acharya *et al.* to characterise hydrophobicity of realistic protein surfaces accurately, this method is based on the fact that hydrophobic probes (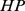), here neutral Lennard-Jones methane-like particles [1], are attracted to hydrophobic patches on the whole. The hydrophobicity of a residue is then defined as the averaged probability that a residue is in contact with a hydrophobic probe. A contact is defined if the minimum distance between an amino-acid residue and a 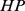 is smaller than 0.4 nm.

We performed eight of these hydrophobic-probe calculations, four on an extended conformation and four on a collapsed conformation (structure 1 in Figure 1(B)). Figures 4(C) and (D) present the result of these hydrophobic-probe calculations for the two conformations. The two barcodes are coloured on an RWG hydrophobicity scale (as Figure 4(B)). Figure 4(E) shows a white-blue scaled barcode plotting the extent of burial of individual residues to the interior upon a full collapse (blue indicating solution exposure in the new conformation, while white colour-coded residues are buried upon collapse). Clearly, the three hydrophobicity maps show that hydrophobicity of the surface of TF changes with conformation and cannot be ascertained accurately from the standard hydrophobicity of individual residues. Secondly, the differences between the hydrophobicity maps on the extended and collapsed conformations can be accounted for by the residue burial map. On the head and N-terminal, the buried residues tend to show a higher hydrophobic character and exposed residues a lesser hydrophobic character. This suggests that certain groups of buried residues on these two domains trap hydrophobic probes, but are unable to do so upon being solvent exposed.

The graphs show that the two arms (residues 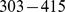) demonstrate the most hydrophobic patches in both collapsed and extended conformations. The patches at residues 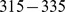 and 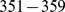 on arm1, and at residues 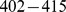 on arm2 are especially prominent. The patches at residues 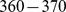 and 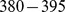 on arm2 display a pronounced hydrophobic behaviour in the collapsed conformation, but not in the extended conformation. This suggests that the interactions with N-terminal induces hydrophobic behaviour in arm2. Additionally, the hydrophobic behaviour of groups of residues at 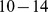, 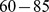 and 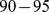 (forming 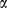-helices of N-terminal) in the extended conformation of Trigger factor is subdued upon the NtA2 collapse, likely because they are buried during the NtA2 collapse. The PPIase domain exhibits a moderate hydrophobic character at several patches of residues: 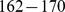, 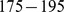 and 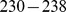. The hydrophobic behaviour is enhanced in the collapsed conformation by creating traps for the probes. Conversely, the hydrophobicity in residues 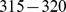 and 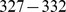 is attenuated because of burial into the interior.

The contact map of NtA2 and HA1 interactions can also be analysed in the terms of hydrophobic patches (see figure 4(F)). The residues from 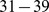 on N-terminal exhibit a high probability of interacting with arm2, but do not contain a hydrophobic patch in either conformation. On the other hand, the residues at 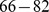, which contain a hydrophobic patch, frequently form contacts with arm2. The corresponding residues on arm2, between 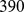 and 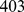, lie outside the strongly hydrophobic patch at residues 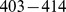. Similarly, the head comprises of a strongly hydrophobic patch between residues 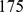 and 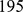 (especially in the collapsed state), which are also involved in multiple contacts with arm1. Another group of residues at 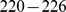 that forms multiple contacts with arm1 contains no hydrophobic patches. In contrast, nearly the entire group of residues on arm1, between 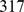 and 337, that form contacts with residues on the head are localised within a strongly hydrophobic patch. These observations suggest that most of the interactions between head and arm1 are hydrophobic, as well as many of those between N-terminal and arm2. However, N-terminal and arm2 also form hydrophilic contacts.

**Figure 5:**
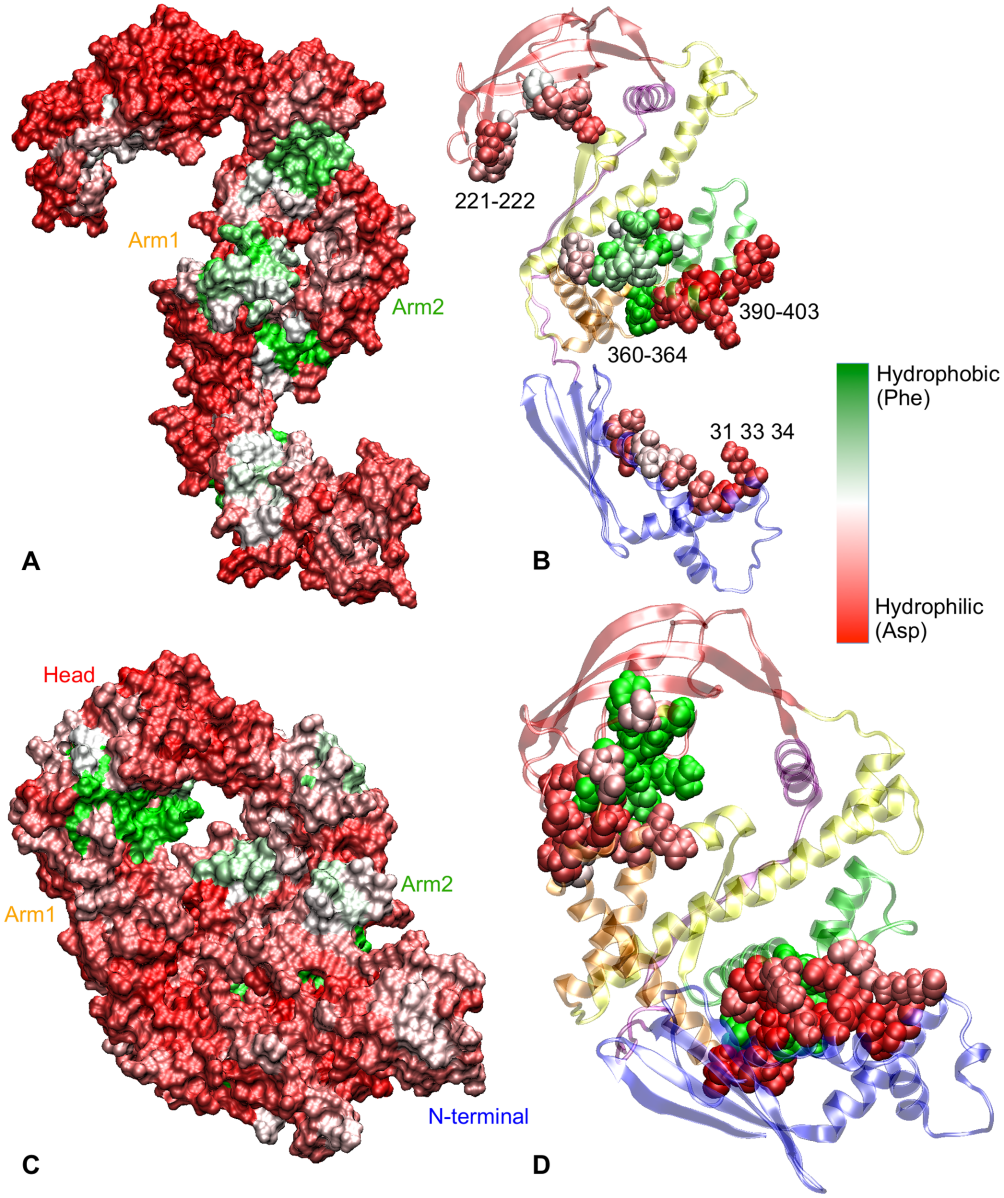
Structural view of hydrophobicity in Trigger factor. (**A**) Surface of Trigger factor colour-coded according to the red-white-green (RWG) hydrophobicity scale for the extended conformation. (**B**) Cartoon representation of Trigger factor with bead-representation (RWG hydrophobicity scale for extended conformation) for the residues involved in inter-domain interactions responsible for stabilisation of the “collapsed” conformation. (**C**) Cartoon representation of Trigger factor’s collapsed conformation with interacting residues represented as beads (RWG hydrophobicity scale for collapsed conformation). (**D**) Surface of Trigger factor (collapsed conformation) colour-coded according to the RWG hydrophobicity scale for collapsed conformation.

Based on these 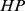 calculations, we visualised the surface hydrophobicity of Trigger factor in the extended and collapsed conformations in Figures 5(A) and (D). The surface of arm1 is dominantly hydrophobic, while the 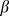-sheet structure of the head also shows a hydrophobic character. On the other hand, N-terminal and arm2 can be expected to show hydrophilic character. The residues involved in inter-domain contacts are shown in Figure 5(B) in cartoon representation of TF’s extended structure. The residues on arm1 that dominate HA1 contacts are strongly hydrophobic, while most of the corresponding residues on the head are also hydrophobic. The residues that form the contacts in NtA2 interaction are dominantly hydrophilic, both in N-terminal as well as in arm2. This confirms the previously drawn implications that while both HA1 and NtA2 collapses are driven by hydrophobic contacts, NtA2 collapse is further stabilised by hydrophilic contacts. Upon collapse, the hydrophobicity of several residue-patches is changed, as is observable from Figure 5(C) that shows the interacting residues as beads in a cartoon representation of TF’s collapsed conformation.

### Summary

To understand the functional dynamics of TF in cytosol or *in vitro* experiments, it is imperative to first understand its structural dynamics in solution. To date, both the solution structure of TF and its dynamics has not been characterised. Our simulations suggest that the solution structure of monomeric TF is highly flexible and differs from the crystal structure. The variations of respective domains from the crystal structure are much more pronounced than the degree of flexibility suggested in literature [20]–[24], [28], [30]. This suggests that the measurement of flexibility of TF is underestimated in literature. We find that this enhanced flexibility facilitates a structural collapse of TF in solution. As the simulations cannot be expected to reach an equilibrium in 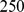 ns, we observe significant heterogeneity in this collapse with a variety of structures that TF can acquire. Still, all simulation trajectories show either semi-collapsed (with HA1 collapse) or fully collapsed (HA1 collapse followed by NtA2 collapse) conformations. The collapse is characterised by structural motions of individual domains, and facilitated by hinge-like behaviour of inter-domain linkers. During the collapse, the molecule displays large structural fluctuations, which are subdued after acquiring the fully collapsed conformation. While we can expect that the structure of TF in solution is different from that in the crystal, the extent and magnitude of the observed flexibility is remarkable. This flexibility appears to vary somewhat with the forcefield employed, as seven out of eight trajectories run with AMBER03 trajectories result in the fully collapsed conformation after 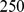 ns, and none of the trajectories run with OPLS-AA parameters do so. On the other hand, the HA1 collapse is common to both forcefields. Slight differences in treatment of charges on individual hydrophilic residues in the two forcefields might affect the probability of NtA2 collapse.

Figure 4(D) shows that many of the distinctly hydrophobic patches coincide with the groups of residues that form inter-domain contacts in the collapse, especially in the case of head and arm1. The same is true for the N-terminal and arm2 interaction, though the more dominantly involved residues (Table 2) lie outside the hydrophobic patches. Together with the hydrogen bond distribution (Figure 3(B)), list of involved residue pairs (Table 1) and the hydrophobicity map for these residue pairs (Figure 5(C)), this observation suggests that both HA1 and NtA2 collapses are triggered mainly by hydrophobic contacts. However, while HA1 collapse is almost entirely dominated by hydrophobic contacts, NtA2 collapse is stabilised predominantly by hydrophilic contacts – hydrogen bonds as well as salt-bridges. We also expect highly hydrophobic patches at the back of N-terminal and arm2, as shown in Figure 5(B), to play a role in triggering the NtA2 collapse. Thus, we can conclude that the structural collapse of TF in solution is driven by the burial of these hydrophobic patches to form the interior of the collapsed conformation, and ultimately stabilised by hydrophilic contacts.

The observed ability of TF to form a variety of non-covalent protein-chaperone interactions – hydrophobic as well as hydrophilic – makes it a promiscuous chaperone with the ability to interact with a variety of proteins and polypeptide chains [11], [24]. Moreover, the structural flexibility displayed by TF, defined by its ability to undergo significant conformational changes, is expected to facilitate the chaperone’s adaptation to accommodate proteins of diverse shapes and sizes [11]. The chaperone may explore its rather large conformational space to accommodate the substrate protein and establish the necessary contacts, comprising of hydrophobic as well as hydrophilic contacts. Consequently, it will be of great interest to investigate whether competing interactions between TF and substrate proteins *in vitro* as well as *in vivo* will limit entry into the collapsed states.

This work gives a new perspective on the dynamics and flexibility of TF in solution. It is also an important step in characterising the surface properties of TF. Finally, our study can also be developed further to provide an insight into the binding mechanism of substrate proteins to cytosolic TF, both *in vitro* as well as *in vivo*.

## Materials and Methods

### Molecular Dynamics

As a starting point for this work, a single TF chain (Chain A) was extracted from the crystal structure (PDB code 1W26). The GROMACS 4.5.1 package was employed for preparation of the system and all-atom MD simulations [45], [46]. Hydrogen atoms were added to the PDB structure for an effective protonation state at pH 7.0, while atomic interactions were defined using AMBER03 [39] (eight MD runs) and OPLS-AA [40] (four MD runs) force fields. Coulombic and van der Waals interactions were treated with a cut-off radius of 1.2 nm, while long-range electrostatic interactions were handled using the PME algorithm (with a mesh size of 0.4 nm). The structure was subsequently relaxed through the steepest descent energy-minimisation algorithm. The structure defined by AMBER03 parameters was solvated in a dodecahedron periodic box (of diameter 13.2 nm) of 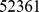 TIP/3P water molecules, and neutralised by adding 81 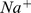 and 58 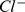 ions (50 mM NaCl); while, the structure defined by OPLS-AA parameters was solvated in dodecahedron box (of diameter 14 nm) of 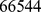 TIP/3P water molecules, and neutralised by adding 150 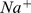 and 127 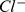 ions (50 mM NaCl). The system was energy-minimised again, and a 50 ps position-restrained (all atoms of TF restrained with force constant of 1000 N/m in each direction) MD run was performed on the obtained *Trigger factor in water* system to equilibrate the positions of water molecules and ions. Please note that a box with diameter 13.2 nm is sufficient to house the extended crystal structure of TF.

To optimise the protein-water interaction energy and investigate the structural dynamics of TF in solution, twelve parallel full system MD runs were performed (with different random starting velocities) for 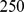 ns at close to room temperature (295 K). Eight of these computations were run with AMBER03 forcefield parameters [39], i.e. four of them with a time step of 2.0 fs (coordinates were written every 2.0 ps) and another set of four with a time-step of 1.5 fs (coordinates were written every 7.5 ps). We used a time step of 1.5 fs in the first set of simulations to guarantee that the time step would not lead to a crash in the simulations. We then switched to the 2.0 fs time step because of its higher efficiency. Of course we still can use our 1.5 fs simulation results, while being able to comment on the lack of an effect of time steps. The last four trajectories were run with OPLS-AA forcefield parameters [40] with a time step of 2.0 fs (coordinates were written every 2.0 ps). The system was coupled with a v-rescale thermostat (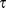 = 0.2) and a Parrinello-Rahman barostat (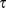 = 1.0 and reference pressure = 1 Bar). The bonds in the protein were constrained using the LINCS algorithm [47].

### Analysis

To analyse the resulting trajectories (comprising of cartesian coordinates of each atom every 2 ps), we employed the analysis tools provided in the GROMACS 4.5.1 package [45]. Distances were calculated between centres of mass of index groups of residues. To quantify structural deviations, root-mean square deviations were calculated and averaged over C-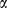 atoms of all residues per time. Sites of fluctuations were estimated through calculation of time-averaged root-mean square deviations of C-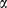 atoms of each amino-acid residue. The crystal structure was used as the reference structure for all analysis.

A hydrogen bond was counted when the distance between a partially charged hydrogen atom (bonded to atom X - nitrogen or oxygen) is within 0.35 nm of an oppositely charged heavy atom (atom Y - oxygen or nitrogen), and the angle formed by X-H-Y is larger than 150°. Due to this rather strict criterion used for the definition of hydrogen bonds in MD simulations, these hydrogen bonds are turned on and off at a high rate. As a result, the distribution of hydrogen bonds over 50 ns windows appears broad and peaks at a lower value of N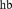 than expected.

To find correlations between order parameters one can compute two-dimensional projections of the trajectories on pairs of order parameters. Such projections are conveniently done by taking the negative logarithm of the histogram 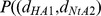, yielding 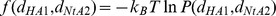, where 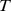 is the temperature, 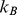 is Boltzmann’s constant, and 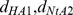 are the two order parameters (i.e. the two pair-wise distances). Note that while 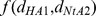 is formally a Landau free energy, we do not claim that the simulations are converged.

To estimate the most likely contact residue pairs involved in the inter-domain contacts, we calculated the probability that a residue pair (in NtA2 and HA1 configurations) has a minimum distance less than 0.4 nm. Minimum distance refers to the minimum distance between any corresponding atoms in a contact pair. The most frequently occurring contact pairs are listed in Tables 1 and 2.

To characterise hydrophobic patches on TF surface, we employed the hydrophobic probes method, as developed by Acharya *et al.* [1], [2]. These hydrophobic probes were defined in AMBER03 forcefield [39] as neutral Lennard-Jones methane-like hydrophobic particles with the following parameters: 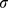 = 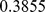 nm, and 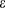 = 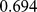 kJ/mol [1]). We introduced 30 of these LJ particles into the system and randomly distributed them around the protein. While position-restraining the heavy atoms of TF, we ran 30 ns long MD simulations - 4 simulations with TF position-restrained in extended conformation, and 4 with TF position-restrained in collapsed conformation (structure 1 in Figure 1). We expect that 30 ns long simulations allowed the hydrophobic particles ample time to explore the hydrophobic patches on TF’s surface. Then, we calculated the minimum distance of 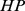 from each amino-acid residue of TF, and plotted histograms (as colour-coded barcodes) for contact probability of 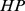 with each residue (i.e., if the minimum distance between an amino-acid residue and 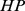 was between 0.2 and 0.4 nm, it was considered as a contact). To estimate the group hydrophobic behaviour, histograms were averaged over sequential groups of 5 residues.

## Supporting Information

File S1The file is divided into three sections: Structural dynamics of TF; Comparisons with NMR study; and GROMOS simulations. The first section illustrates structural deviations of trigger factor in solution, including those of individual domains, in all trajectories. The second section qualitatively compares our results to the NMR study conducted by Yao *et al.* [30]. The third section shows and discusses the results of 250 ns long simulations run with GROMOS43a1 parameters. **Figure S1:** Structural dynamics of Trigger factor in solution. **Figure S2:** Structural deviations of respective domains over time with respect to their conformations in crystal structure. **Figure S3:** Conservation of 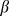-sheet structure between residues K127I129 and T418T422. **Figure S4:** Results from 250 ns long MD simulations run with GROMOS43a1 forcefield parameters.(PDF)
