# Supplementary material for: Hydrophobic Collapse of Trigger Factor Monomer in Solution
Source: PLoS One. 2013 Apr 2;8(4):e59683. doi: 10.1371/journal.pone.0059683 (PMC3615003; doi:10.1371/journal.pone.0059683)
Supplement: File S1 — The file is divided into three sections: Structural dynamics of TF; Comparisons with NMR study; and GROMOS simulations. The first section illustrates structural deviations of trigger factor in solution, including those of individual domains, in all trajectories. The second section qualitatively compares our results to the NMR study conducted by Yao et al. [30]. The third section shows and discusses the results of 250 ns long simulations run with GROMOS43a1 parameters. Figure S1: Structural dynamics of Trigger factor in solution. Figure S2: Structural deviations of respective domains over time with respect to their conformations in crystal structure. Figure S3: Conservation of -sheet structure between residues K127I129 and T418T422. Figure S4: Results from 250 ns long MD simulations run with GROMOS43a1 forcefield parameters. (PDF) [file pone.0059683.s001.pdf]

SI appendix for

Hydrophobic Collapse of Trigger Factor Monomer in Solution

Kushagra Singhal[1], Jocelyne Vreede[1], Alireza Mashaghi[2], Sander J. Tans[2], Peter G. Bolhuis[1,*]

1 van 't Hoff Institute of Molecular Sciences, Amsterdam, The Netherlands

2 Department of Systems Biophysics, AMOLF Institute, Amsterdam, The Netherlands

$*$ **E-mail: P.G.Bolhuis@uva.nl**

Structural Dynamics of Trigger Factor

Figure 1A shows the radius of gyration graphs of all 12 trajectories over time. We see a clear segregation of collapse, and hence a disparity in resultant compactness of structures. Five trajectories show a higher final value of radius of gyration, and can be grouped together to be represented by SC because they have been visually identified to show a semi-collapse. The second set of trajectories shows a full collapse and higher compactness with lower final values of radius of gyration. Based on further visual analysis and comparison with RMSD plot (shown in figure 1B), we have divided them into two groups: six of them are represented by FC; and the one with lowest final value of radius of gyration – and highest magnitudes of RMS deviation (fig. 1B) and fluctuation (figure 1C plots time-averaged RMS fluctuations over complete trajectories), shown in the same colour across the plots – is represented by CD. Figure 1D presents time-averaged RMS fluctuations for each trajectory over the last 20 ns to compare the magnitude of fluctuations during and after the collapse in each trajectory. We notice that while there is a significant drop (by almost a factor of 3) in fluctuations for trajectories represented by FC and CD, fluctuations in semi-collapsed (SC) trajectories do not get attenuated as much.

Figure 2 shows graphs for structural deviations in individual domains. We observe, on average, relatively small deviations in the structures of domain from their crystal conformations, confirming our hypothesis that the collapse of TF is focused in the hinged movements of individual domains rather than deviations within them.

Comparison with NMR Study

Further, the NMR study by Yao *et al.* [1] showed that residues K127–I129 form a parallel beta sheet with residues T418–T422. Figure 3 shows that, in our simulations, this parallel β-sheet structure is conserved despite the collapse and formation of a structure more compact than the X-ray structure of TF.

GROMOS Simulations

We also performed 250 ns long (with a time step of 2.0 fs) MD simulations (20 mM NaCl) on Trigger factor using the GROMOS43a1 forcefield [2](and SPC water model). They showed a structural collapse with the formation of compact structures – only one out of four trajectories resulted in an extended conformation (fig. 4). These results were not presented in the paper, because the salt (NaCl) concentration used was different (20 mM, as opposed to the 50 mM concentration used in the rest of simulations). Moreover, secondary structure losses in Gromos were slightly higher than expected; we attributed these losses to the low effective force constant of backbone H-bonds in GROMOS43a1 (please see Table 3 in [3])

References

1. Yao Y, Bhabha G, Kroon G, Landes M, Dyson HJ (2008) Structure discrimination for the C-terminal domain of Escherichia coli trigger factor in solution. Journal of biomolecular NMR 40: 23–30.

2. Van Gunsteren FW, Billeter SR, Eising AA, Hünenberger PH, Krüger P, et al. (1996) Biomolecular Simulation: The GROMOS96 Manual and User Guide. Zürich, Switzerland: Vdf Hochschulverlag AG an der ETH Zürich, 1–1042 pp.

3. Rueda M, Ferrer-Costa C, Meyer T, Pérez A, Camps J, et al. (2007) A consensus view of protein dynamics. Proceedings of the National Academy of Sciences of the United States of America 104: 796–801.

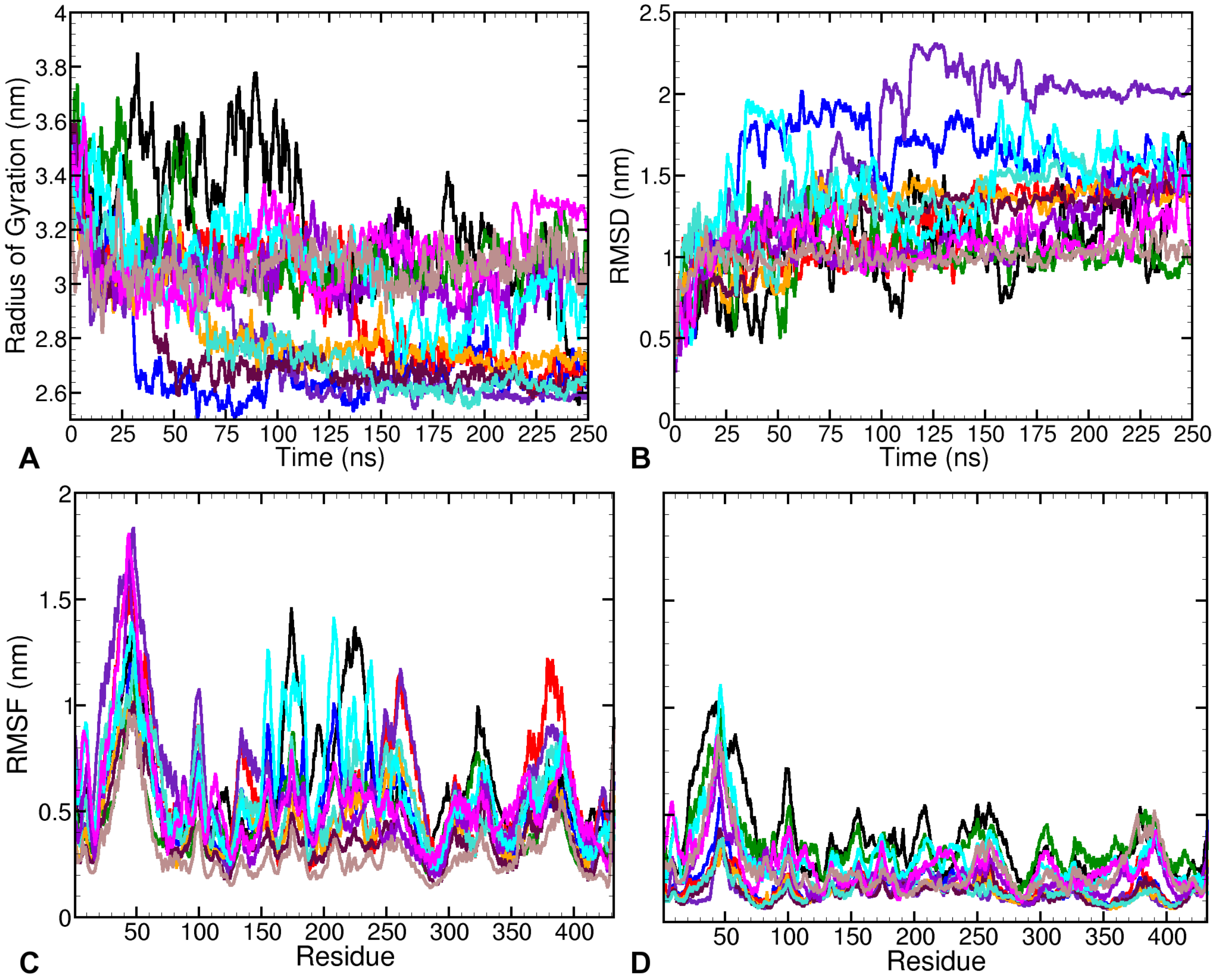

Figure 1. Structural dynamics of Trigger factor in solution (A) Collapse of Trigger factor represented by decrease of radius of gyration of the protein over time. We can categorise the resulting trajectories into roughly 2 groups (represented by FC and SC, respectively) based on the magnitude of final radius of gyration. **(B)** Structural deviations over time given by root mean square deviations from the crystal structure for all 12 trajectories. **(C)** Root mean square fluctuations for all trajectories averaged over the whole simulation. **(D)** Root mean square fluctuations for all trajectories averaged over the last 20 ns of the simulations, indicating a significant structural stability induced by collapse.

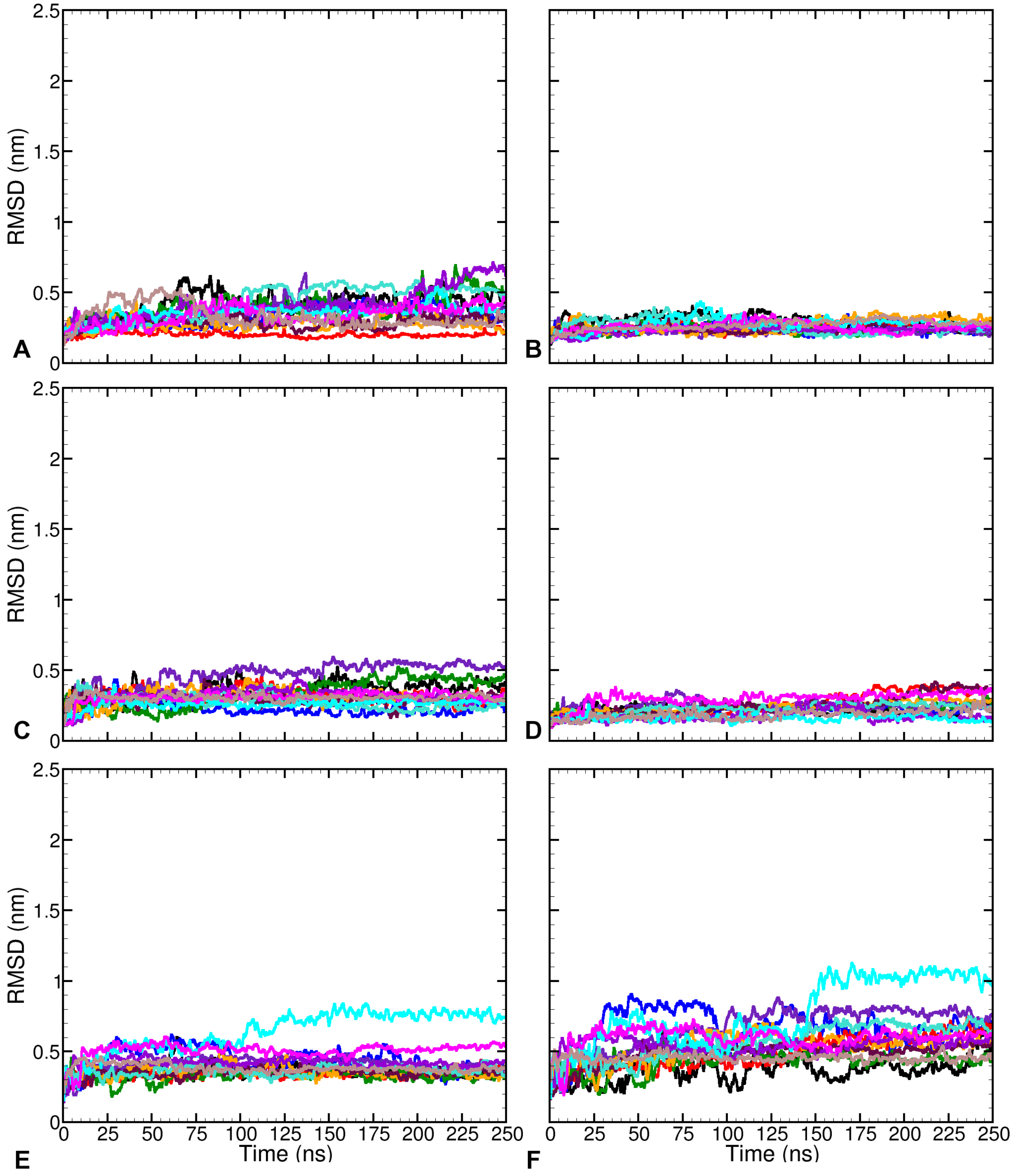

Figure 2. Structural deviations of respective domains over time, with respect to their conformations in crystal structure: (A) N-terminal. **(B)** Head pr PPIase domain. **(C)** Arm1. **(D)** Arm2. **(E)** HA1-linker. **(F)** Linker between N-terminal and Head. The figures show small structural deviations in respective domains from their initial conformations.

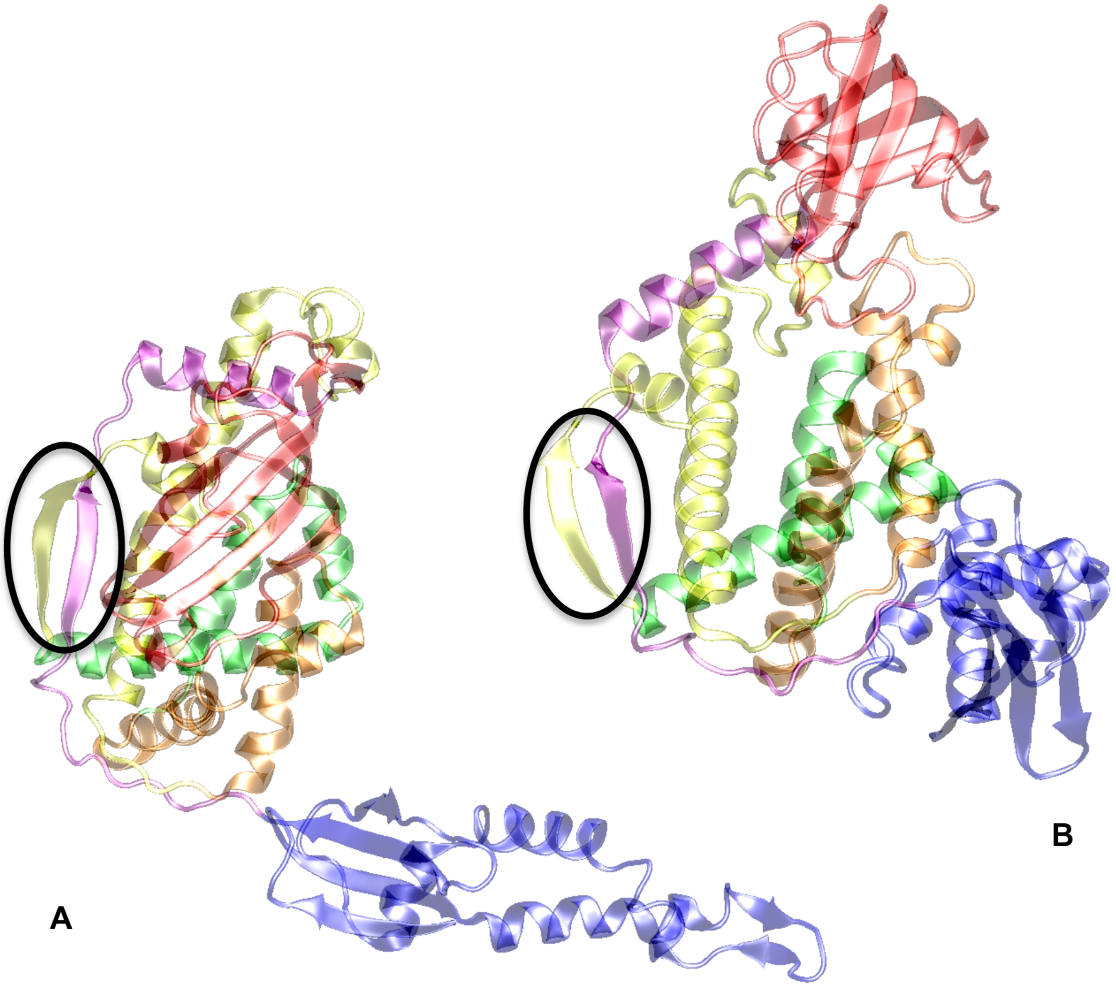

Figure 3. Conservation of β-sheet structure between residues K127–I129 and T418–T422 in the final: **(A)** Semi-collapsed structure; **(B)** Fully collapsed structure.

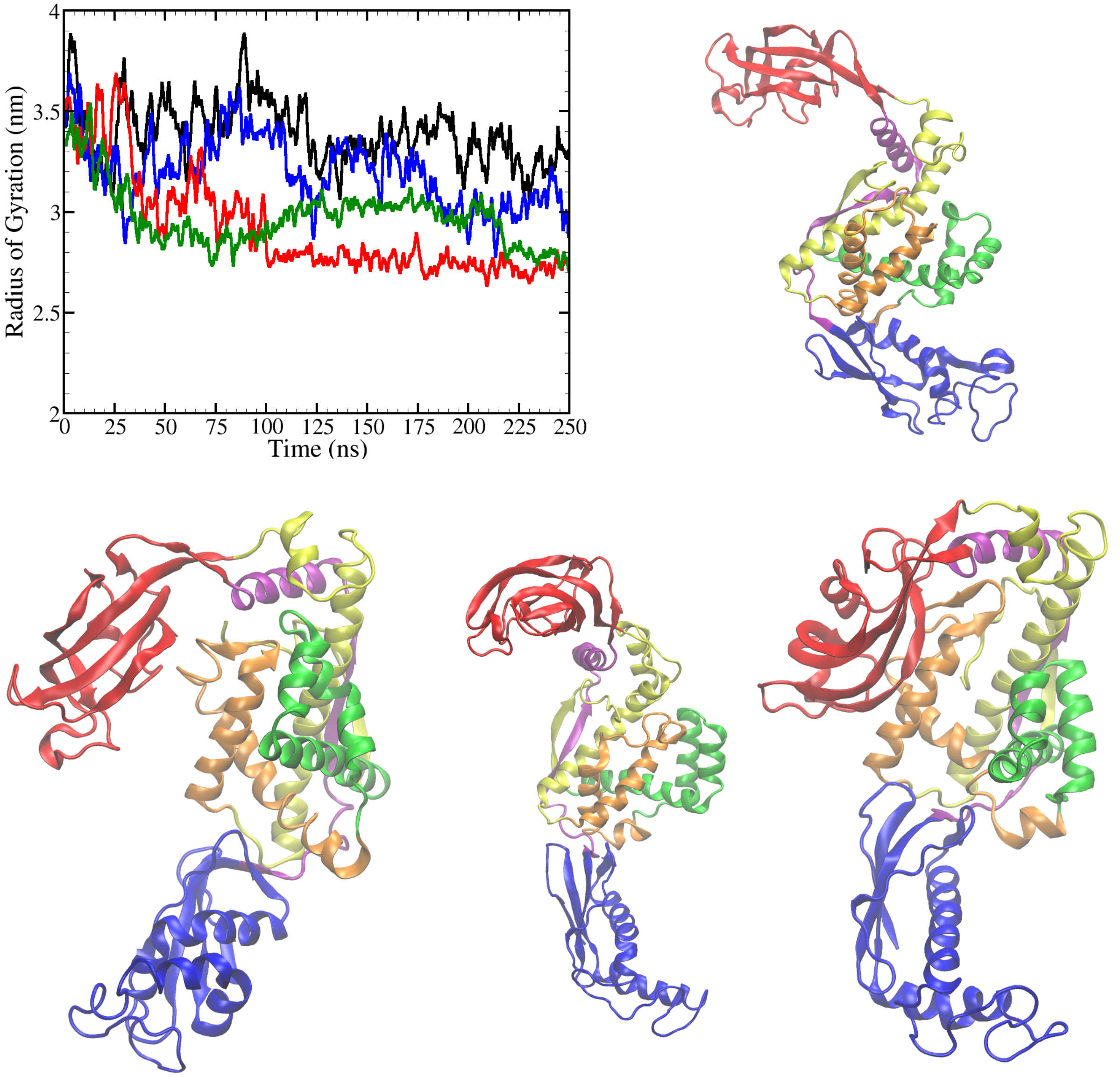

Figure 4. Results from 250 ns long MD simulations run with GROMOS43a1 forcefield parameters: Plot of the variation of radius of gyration of TF over time. The four resultant structures.
